# Development of a practical synthesis of etravirine via a microwave-promoted amination

**DOI:** 10.1186/s13065-018-0504-4

**Published:** 2018-12-20

**Authors:** Da Feng, Fenju Wei, Zhao Wang, Dongwei Kang, Peng Zhan, Xinyong Liu

**Affiliations:** Department of Medicinal Chemistry, Key Laboratory of Chemical Biology (Ministry of Education), School of Pharmaceutical Sciences, Shandong University, 44 West Culture Road, Ji’nan, 250012 Shandong People’s Republic of China

**Keywords:** Etravirine, Microwave-promoted, Amination, Synthesis

## Abstract

**Background:**

Etravirine (ETV) was approved as the second generation drug for use in individuals infected with HIV-1 in 2008 by the U.S. FDA with its unique antiviral activity, high specificity, and low toxicity. However, there are some shortcomings of the existing synthetic routes, such as the long reaction time and poor yield.

**Results:**

This article describes our efforts to develop an efficient, practical, microwave-promoted synthetic method for one key intermediate of ETV, which is capable of being operated on a scale-up synthesis level. Through this optimized synthetic procedure, the amination reaction time decreased from 12 h to 15 min and the overall yield improved from 30.4 to 38.5%.

**Conclusion:**

Overall, we developed a practical synthesis of ETV via a microwave-promoted method, and the synthetic procedure could be amenable to scale-up, and production costs could be significantly lowered.

## Background

HIV-1 non-nucleoside reverse transcriptase inhibitors (NNRTIs) represent a potent and promising antiviral agents that specifically target the HIV-1 reverse transcriptase (RT), the primary target for anti-HIV drugs. The NNRTIs were the major components of highly active antiretroviral therapy (HAART). However, the rapid emergence of drug-resistant HIV-1 strains limited their clinical use [1–4]. Etravirine, 2,4-[[6-amino-5-bromo-2-[(4-cyanophenyl)amino]-4-pyrimidinyl]oxy]-3,5-dimethylbenzonitrile, a second-generation drug of the diarylpyrimidine-based NNRTIs, was approved in 2008 by the U.S. Federal Drug Administration (FDA) for use in HAART [5]. Etravirine is a well-tolerated NNRTI with higher genetic barrier for resistance and good safety profiles compared to the first-generation NNRTIs [6]. However, there are some shortcomings of the existing synthetic routes, such as the long reaction time and poor yield, which lead to the expensive price of etravirine. Therefore, an efficient synthesis of etravirine holds great potential in both scientifically and socially.
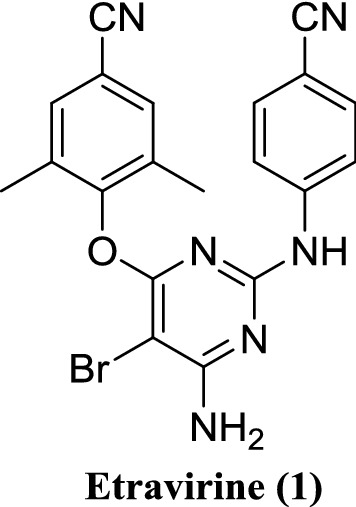


## Medicinal chemistry synthesis of etravirine

**Scheme 1:**
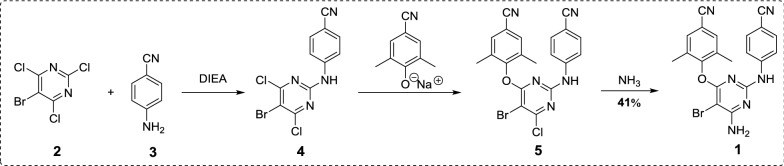
Synthesis of etravirine with 5-bromo-2,4,6-trichloropyrimidine (**2**) as starting material [6]

**Scheme 2:**
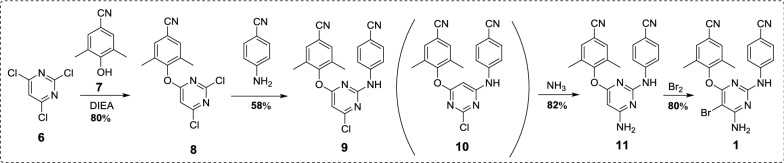
Synthesis of etravirine with 2,4,6-trichloropyrimidine (**6**) as starting material [7]

**Scheme 3:**
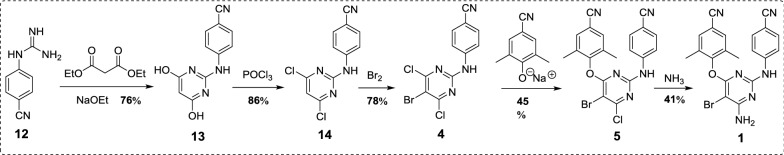
Synthesis of etravirine with 4-guanidinobenzonitrile (**12**) as starting material [8]

**Scheme 4:**
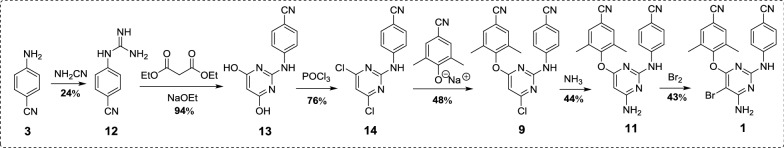
Synthesis of etravirine with 4-aminobenzonitrile (**3**) as intermediate [9]

The synthetic routes of etravirine disclosed are outlined in Schemes 1, 2, 3 and 4, which is mainly divided into two methods: (1) Method 1: The halogenated pyridines (**2** or **6**) are used as starting materials (Schemes 1, 2) [6, 7]; (2) Method 2: 4-guanidinobenzonitrile (**12**) is selected as starting material or intermediate (Schemes 3, 4) [8, 9].

In Scheme 1, the starting material 5-bromo-2,4,6-trichloropyrimidine (**2**) was treated with 4-aminobenzonitrile (**3**) in refluxing dioxane to give the intermediate **4**. Treatment of **4** with 4-hydroxy-3,5-dimethylbenzonitrile in *N*-methylpyrrolidone afforded the key intermediate **5**. Then etravirine was obtained by the ammonification reaction of intermediate **5** with ammonia under the condition of high pressure and high temperature.

In another synthetic route (Scheme 2), the starting material 2,4,6-trichloropyrimidine (**6**) was treated with 4-hydroxy-3,5-dimethylbenzonitrile (**7**) under the weakly alkaline condition yield the intermediate **8**. Then **8** reacted with 4-aminobenzonitrile (**3**) provided the intermediate **9** and by-product **10**, which was separated from each other by recrystallization. Then etravirine was obtained by the ammonification and bromination of intermediate **9** successively. The yield of the overall route can up to 30.4%.

In Scheme 3, etravirine was obtained with the 4-guanidinobenzonitrile (**12**) as starting material. Firstly, **12** was cyclized with diethylmalonate in the presence of sodium ethoxide in ethanol to give the intermediate **13**, which was subsequently treated with POCl_3_ to form the corresponding derivative **14**. Then the bromination of **14** afforded the intermediate **4**, which passed through four successive reactions (nucleophilic substitution with the sodium salt of **7**, and ammonification) to give etravirine. In Scheme 4, the synthesis route very similar to that in Scheme 3. The more commercially available 4-aminobenzonitrile (**3**) was used as starting material in this route. Besides, the sequence of the last three steps in Scheme 4 (nucleophilic substitution, ammonification and bromination) is distinct from those in Scheme 3.

Taken together, in the above synthesis methods of etravirine, problems like the following still exist: (1) The starting materials are difficult to obtain (exemplified by compound **2**); (2) In the route employing 4-guanidinobenzonitrile as starting material or intermediate, the overall yield is low; (3) The longer amination reaction time and lower yield of the overall route when halogenated pyridine was used as starting material. Therefore, there have an urgent need to find more efficient and practicable methods in the pharmaceutical industry to synthesize etravirine and its intermediates. Comparative analysis the existing routes described above, the route in Scheme 2 has advantages of the accessibility of raw materials and the simplicity of synthetic steps. Inspired by the route in Scheme 2 and considering its deficiency, we became interested in designing a more efficient synthesis through optimizing the amination method with the aim to increase the overall yield of the route and shorten the longer amination reaction time.

## Results and discussions

Since Gedye and Giguere published their first articles about microwave-assisted syntheses in household microwave ovens in 1986 [10, 11], the microwave-assisted synthesis method have attracted an increasing number of chemists’ attention for its high efficiency in chemical process. The method have been used in many fields successfully. Considering the longer amination time of the existing process route, we attempt to apply this efficient method in the amination reaction for the purpose of reducing reaction time and improving the yield.

**Scheme 5:**
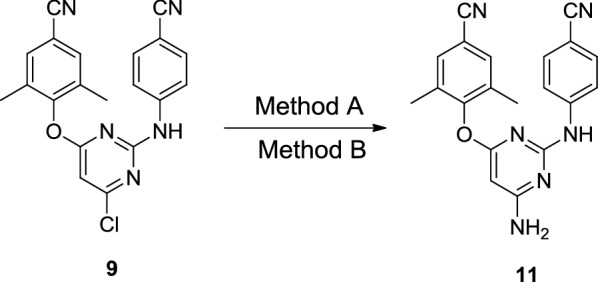
Amination reaction of the intermediate **9**

In the preliminary study, we conducted the reaction in an autoclave as the conventional synthesis [5] (Scheme 5). The amination reaction performed very well as the literature reported and the yield ranged from 82.7 to 83.6%. Then we attempted the reaction in the microreactor. When we conducted our first attempt, dioxane, acetonitrile and tetrahydrofuran was used as solvent. The results were frustrated and there no desired product was obtained. We speculated that the poor solubility of the intermediate **9** in these solvent lead to the failure of the reaction. Then some good dissolving solvent of **9** were chosen, such as dimethylformamide (DMF), dimethylsulfoxide (DMSO) and *N*-methylpyrrolidone (NMP). The results were depicted in Table 1, the reaction conducted very well in all the three solvent with moderate to good yield compared to our preliminary attempt. The results demonstrated that the reaction have the best yield in *N*-methylpyrrolidone, so it was selected as solvent for the further optimization of the microwave reaction. Further investigation of the amination reaction mainly focus on the amination temperature and reaction time (Table 2). We can conclude that the yield was improved with the increased reaction time and temperature. But there have decreasing tendency of the yield when the temperature above 130 °C and reaction time more than 15 min. After an orthogonal experiment, the optimized conditions of the amination reaction was determined as follows: in the microwave reactor with *N*-methylpyrrolidone as solvent and reacted in 130 °C for 15 min. The yield of amination reaction can up to 85.6%, which was higher than that of the conventional synthesis method (83.6%).

**Table 1:** Optimization of reaction conditions

Solvent	Method^a^	Temperature (°C)	Time	Pressure (psi)	Yield %
Dioxane	A	120	12 h	–	83.5
CH_3_CN	A	120	12 h	–	82.7
NMP	A	120	12 h	–	83.6
Dioxane	B	120	30 min	130	0
CH_3_CN	B	120	30 min	130	0
THF	B	120	30 min	130	0
DMF	B	120	30 min	128	67.7
DMSO	B	120	30 min	127	72.1
NMP	B	120	30 min	127	81.4

^a^Method A: Conventional synthesis: 25% aq ammonia, autoclave, 120 °C, 12 h; Method B: Microwave-assisted synthesis: 25% aq ammonia, 10–30 min, 120–150 °C

**Table 2:** Optimization of amination reaction conditions

	Yield/%					
Tem./°C:	110	120	125	130	135	140
*Time/min*						
5	32.4	42.1	45.3	43.2	45.2	47.6
10	46.7	64.5	74.2	71.2	76.3	73.6
15	64.4	78.3	82.4	85.6	85.3	85.5
20	74.3	80.8	82.6	85.3	85.6	85.1
25	80.4	81.2	83.1	85.7	83.1	84.3
30	82.5	81.4	82.1	84.2	84.2	84.1

## Discussion

**Scheme 6:**
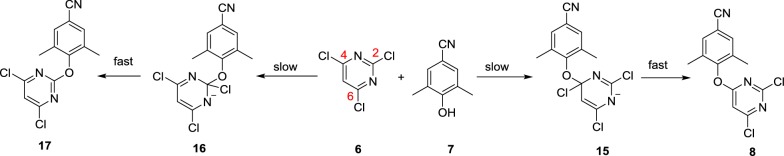
The reaction mechanism of the intermediate **6** and **7**

The first step of the process route is a typical S_N_Ar (addition–elimination) process (Scheme 6). Treatment of equimolar amounts of 4-hydroxy-3,5-dimethylbenzonitrile (**7**) with 2,4,6-trichloropyrimidine (**6**) in the presence of potassium carbonate may be afford two mono-substituted products **8** and **17** at the position of C_4_–Cl and C_2_–Cl of the staring material **6**. But the mono-substituted product **8** were obtained with excellent yields, for the reason that there exist a selectivity between C_4_–Cl and C_2_–Cl of compound **6** [12]. On account of the S_N_Ar (addition–elimination) process is thermodynamical control, so the product depend on the stabilization of the intermediate Meisenheimer complex. Compared to the Meisenheimer intermediate **16** where the ring nitrogen ortho to the tetrahedral carbon, the intermediate **15** with the *para*-quinoid structure can be better bear the negative charge and more stabilization [12], which gives reasonable account for the single mono-substituted products **8**.

## Conclusions

**Scheme 7:**
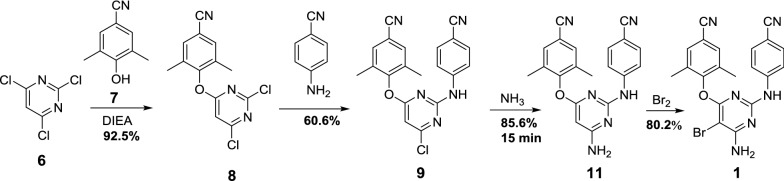
Synthetic route and yield of etravirine

Etravirine is an essential medicine for the treatment of HIV, which is still inaccessible to millions of people worldwide. To overcome the disadvantageous issues in the existing synthetic methods of etravirine, an efficient and practical synthetic method was optimized in this article. The synthesis was achieved using a linear approach starting from 2,4,6-trichloropyrimidine through a sequence of nucleophilic substitution, ammonification and bromination (Scheme 7). The microwave-promoted amination is the most critical step of this route, and it shorten the amination reaction time from 12 h to 15 min. Moreover, the overall yield of the synthetic route is improved from 30.4 to 38.5% over 4 linear steps. To the best of our knowledge, this is the highest yield for etravirine that has been reported. Moreover, all the synthetic process does not require purification by column chromatography, and the formation of impurities could be suppressed very well. Accordingly, the synthetic procedure could be amenable to scale-up, and production costs could be significantly lowered through this microwave-promoted method.

## Experimental section

All melting points were determined on a micro melting point apparatus and are uncorrected. ^1^H-NMR spectra were obtained on a Bruker Avance 400 NMR spectrometer in the indicated solvents. Chemical shifts are expressed in δ units and TMS as internal reference. Mass spectra was taken on a LC Autos ampler Device: Standard G1313A instrument. TLC was performed on Silica Gel GF254 for TLC (Merck) and spot was visualized by iodine vapours or irradiation with UV light (λ = 254 nm). The microwave reaction was conducted on a CEM Discover (0–600 W, 2450 MHz) instrument and the conventional high pressure reaction was performed on Parr 4590 instrument. Concentration of the reaction solutions involved the use of rotary evaporator at reduced pressure.

### 4-[(2,6-Dichloro)-4-pyrimidinyloxy]-3,5-dimethylbenzonitrile (**8**)

2,4,6-Trichloropyrimidine **6** (110 mmol, 20.0 g), diisopropylethylamine (132 mmol, 17.0 g) and 3,5-dimethyl-4-hydroxybenzonitrile **7** (110 mmol, 16.2 g) were dissolved in 1,4-dioxane (100 mL) and the mixed solution was heated at 70 °C for 2 h. After the reaction mixture was brought to 10–15 °C, 200 mL water was poured into the mixture and stirred for another 30 min, filtrated. Then the wet cake was dried at 55–60 °C under vacuum to give the intermediate **8** as white solid with a yield of 92.5%. ^1^H NMR (400 MHz, DMSO-*d*_*6*_, ppm) δ: 7.76 (2H, s, C_3_,C_5_–Ph–H), 7.64 (1H, s, pyrimidine-H), 2.12 (6H, s, CH_3_). ESI–MS: m/z 294.28(M+1). C_13_H_9_Cl_2_N_3_O (293.01), mp: 207–209 °C.

### 4-[[6-Chloro-2-[(4-cyanophenyl)amino]-4-pyrimidinyl]oxy]-3,5-dimethylbenzonitril (**9**)

Compound **8** (68 mmol, 20.0 g) and 4-aminobenzonitrile (68 mmol, 8.0 g) were dissolved in *N*-methylpyrrolidone (100 mL) at 0–5 °C, then the solution was added potassium *tert*-butoxide (136 mmol, 15.3 g) over a period of 30 min and stirred for another 2 h at 0–5 °C. Then the mixture was added to 500 mL water slowly and the resulting precipitate was filtered. The obtained residue was suspended in water (200 mL) and acidified to pH 6–7 with 3 M hydrochloric acid solution, filtered and dried at 55–60 °C under vacuum to give the crude product, which was purified by ethyl acetate treatments (2 × 200 mL) at 70 °C for 30 min followed by filtration at 10 °C and washing the cake with 20 mL of chilled ethyl acetate. Then the wet cake was finally dried at 50 °C under vacuum to give the intermediate **9** as white solid with a yield of 60.6%. ^1^H NMR (400 MHz, DMSO-*d*_*6*_, ppm) δ: 10.56 (1H, s, NH), 7.79 (2H, s, C_3_,C_5_–Ph′–H), 7.45–7.51 (4H, m, Ph–H), 6.93 (1H, s, pyrimidine-H), 2.13 (6H, s, CH_3_). ESI–MS: m/z: 376.5 (M+1), 393.3 (M+18), 398.4 (M+23). C_20_H_14_ClN_5_O (375.09), mp: 277–279 °C.

### 4-[[6-Amino-2-[(4-cyanophenyl)amino]-4-pyrimidinyl]oxy]-3,5-dimethylbenzonitrile (**11**)

A mixture of **9** (5.3 mmol, 2.0 g), 25% aq ammonia (15 mL), and *N*-methylpyrrolidone (20 mL) was put into a microwave reactor and set the condition for 130 °C, 15 min. In the reaction process, the pressure can up to 135 psi. After the reaction mixture was brought to 5–10 °C, 100 mL water was added to this solution followed stirring another 30 min. The generated solid was filtered, washed with 100 mL of water and dried at 45–50 °C to give the crude intermediate **11** as white solid with a yield of 85.6%. ^1^H NMR (400 MHz, DMSO-*d*_*6*_, ppm) δ: 9.57 (1H, s, NH), 7.73 (2H, s, C_3_,C_5_–Ph′–H), 7.65 (2H, d, *J* = 8.0 Hz, C_3_,C_5_–Ph–H), 7.46 (2H, d, *J* = 8.0 Hz, C_2_,C_6_–Ph–H), 6.80 (2H, s, NH_2_), 5.47 (1H, s, pyrimidine-H), 2.12 (6H, s, CH_3_). ESI–MS: m/z 357.4 (M+1), 379.5 (M+23). C_20_H_16_N_6_O (356.14), mp: 283–286 °C.

### Etravirine (**1**)

To a cooled solution of **11** (8.4 mmol, 3.0 g) in DCM (30 mL) at 0–5 °C was added bromine solution (9.4 mmol, 1.5 g in 8 mL of DCM). The reaction was stirred at this temperature for 5 h. Then the mixed solution was diluted with water (50 mL) and basified with 4 M NaOH solution at pH 9–10. The pH of the reaction was maintained between 8 and 9 over a period of another 1 h by adding 4 M NaOH solution and sodium metabisulphite solution. Then the obtained solid was filtered, washed with water (30 mL), and dried at 55–60 °C temperature under vacuum to get crude product, which was following dissolved in methanol (40 mL) at 55–60 °C and treated with activated charcoal. After charcoal clarification, methanol was distilled out, and the residue was recrystallized in ethyl acetate. The crystal was filtered and dried at 55–60 °C under vacuum to give etravirine with a yield of 80.2%. ^1^H NMR (400 MHz, DMSO-*d*_*6*_, ppm) δ: 9.60 (1H, s, NH), 7.75 (2H, s, C_3_,C_5_-Ph’-H), 7.54 (2H, d, *J* = 8.0 Hz, C_3_,C_5_–Ph–H), 7.43 (2H, d, *J* = 8.0 Hz, C_2_,C_6_–Ph–H), 7.13 (2H, s, NH_2_), 2.12 (6H, s, CH_3_). ESI–MS: m/z: 435.4 (M+1), 427.4 (M+3), 457.4 (M+23). C_20_H_15_BrN_6_O (434.05), mp: 254–256 °C.
